# 7,9-Dimethyl-5-phenyl­sulfonyl-5*H*-benzo[*b*]carbazole

**DOI:** 10.1107/S1600536808024380

**Published:** 2008-08-06

**Authors:** G. Chakkaravarthi, V. Dhayalan, A. K. Mohanakrishnan, V. Manivannan

**Affiliations:** a Department of Physics, CPCL Polytechnic College, Chennai 600 068, India; b Department of Organic Chemistry, University of Madras, Guindy Campus, Chennai 600 025, India; c Department of Physics, Presidency College, Chennai 600 005, India

## Abstract

In the title compound, C_24_H_19_NO_2_S, the mean plane of the benzo[*b*]carbazole ring system makes a dihedral angle of 79.26 (5)° with the phenyl ring. The S atom is in a distorted tetra­hedral configuration. The crystal structure exhibits weak C—H⋯O and C—H⋯π inter­actions.

## Related literature

For related literature, see: Allen *et al.* (1987 ▶); Chakkaravarthi *et al.* (2007 ▶, 2008 ▶); Diaz *et al.* (2002 ▶); Etter *et al.* (1990 ▶); Friend *et al.* (1999 ▶); Govindasamy *et al.* (1998 ▶); Hökelek *et al.* (1998 ▶); Hosomi *et al.* (2000 ▶); Itoigawa *et al.* (2000 ▶); Ramsewak *et al.* (1999 ▶); Rodriguez *et al.* (1995 ▶); Sankaranarayanan *et al.* (2000 ▶); Tachibana *et al.* (2001 ▶); Zhang *et al.* (2004 ▶).
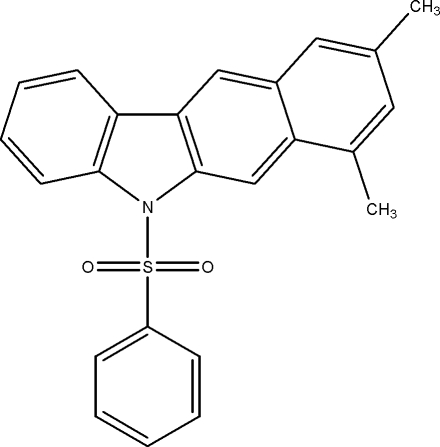


## Experimental

### 

#### Crystal data

C_24_H_19_NO_2_S
*M* *_r_* = 385.46
Orthorhombic, 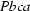

*a* = 13.9260 (6) Å
*b* = 10.1995 (5) Å
*c* = 27.3014 (14) Å
*V* = 3877.8 (3) Å^3^
*Z* = 8
Mo *K*α radiation
μ = 0.19 mm^−1^
*T* = 295 (2) K
0.30 × 0.20 × 0.16 mm

#### Data collection

Bruker Kappa APEXII diffractometer
Absorption correction: multi-scan (*SADABS*; Sheldrick, 1996 ▶) *T*_min_ = 0.940, *T*_max_ = 0.971
26413 measured reflections
5626 independent reflections
3490 reflections with *I* > 2σ(*I*)
*R*_int_ = 0.038

#### Refinement

*R*[*F* ^2^ > 2σ(*F* ^2^)] = 0.051
*wR*(*F* ^2^) = 0.157
*S* = 1.04
5626 reflections
255 parameters
H-atom parameters constrained
Δρ_max_ = 0.68 e Å^−3^
Δρ_min_ = −0.31 e Å^−3^

### 

Data collection: *APEX2* (Bruker, 2004 ▶); cell refinement: *APEX2*; data reduction: *APEX2*; program(s) used to solve structure: *SHELXS97* (Sheldrick, 2008 ▶); program(s) used to refine structure: *SHELXL97* (Sheldrick, 2008 ▶); molecular graphics: *PLATON* (Spek, 2003 ▶); software used to prepare material for publication: *SHELXL97*.

## Supplementary Material

Crystal structure: contains datablocks I, global. DOI: 10.1107/S1600536808024380/bt2759sup1.cif

Structure factors: contains datablocks I. DOI: 10.1107/S1600536808024380/bt2759Isup2.hkl

Additional supplementary materials:  crystallographic information; 3D view; checkCIF report

## Figures and Tables

**Table 1:** Hydrogen-bond geometry (Å, °)

*D*—H⋯*A*	*D*—H	H⋯*A*	*D*⋯*A*	*D*—H⋯*A*
C8—H8⋯O1	0.93	2.37	2.945 (3)	120
C21—H21⋯O2	0.93	2.34	2.935 (2)	122
C8—H8⋯*Cg*1^i^	0.93	3.00	3.859	155
C11—H11⋯*Cg*2^ii^	0.93	2.90	3.693	144

Symmetry codes: (i) 
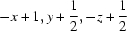
; (ii) 
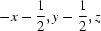
. *Cg*1 and *Cg*2 are the centroids of the C7–C12 and C15–C20 rings, respectively.

**Fig. 1.**
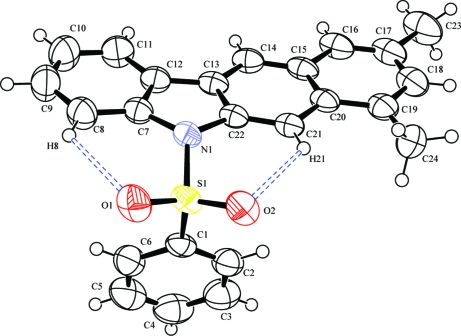
The molecular structure of (I), with atom labeling scheme. Displacement ellipsoids are drawn at 50% probability level. H atoms are presented as a small spheres of arbitrary radius. Intramolecular H-bonds are shown as dashed lines.

**Fig. 2.**
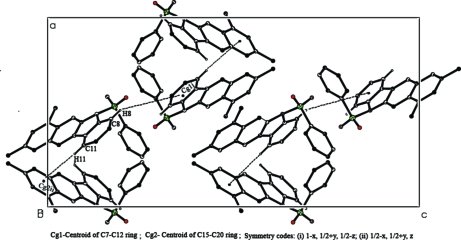
The crystal structure of (I), viewed down the b axis. C—H···π interactions are shown as dashed lines For the sake of clarity, H atoms not involved in interaction have been omitted.

## supplementary crystallographic information

### Comment

Carbazole and its derivatives have become quite attractive compounds owing to their applications in pharmacy and molecular electronics. It has been reported that carbazole derivatives possess various biological activities, such as antitumor (Itoigawa *et al.*, 2000), antioxidative (Tachibana *et al.*, 2001), anti-inflammatory and antimutagenic (Ramsewak *et al.*, 1999). Carbazole derivatives also exhibit electroactivity and luminescence properties and are considered to be potential candidates for electronic such as colour displays, organic semiconductor lasers and solar cells (Friend *et al.*, 1999). These compounds are thermally and photochemically stable, which makes them useful materials for technological applications. For instance, the carbazole ring is easily funtionalized and covalently linked to other molecules (Diaz *et al.*, 2002). This enables its use as a convenient building block for the design and synthesis of molecular glasses, which are widely studied as components of electroactive and photoactive materials (Zhang *et al.*, 2004).

Against this background, and in order to obtain detailed information on molecular conformations in the solid state, X-ray studies of the title compounds (I) have been carried out. X-Ray analysis confirms the molecular structure and atom connectivity for (I), as illustrated in Fig. 1. The benzocarbazole ring is planar, with bond distances and angles comparable to those reported similiar structures (Hökelek *et al.*, 1998; Hosomi *et al.*, 2000). The mean planes of the benzo(b)carbazole and phenyl rings form a dihedral angle of 79.26 (5)°. The N1—S1—C1 plane is almost orthogonal to carbazole ring [dihedral angle 87.20 (7)°] and phenyl ring [dihedral angle 88.44 (7)°]. The best plane of pyrrole ring N1/C7/C12/C13/C22 subtends a dihedral angle of 28.11 (11)° with sulfonyl group.

The average S—O, S—C, and S—N distances are 1.418, 1.760 and 1.664 Å, respectively, in (I); these are comparable as observed in similiar structures (Chakkaravarthi *et al.*, 2007; Sankaranarayanan *et al.*, 2000). The N—C bond lengths, namely N1—C7 and N1—C22 [1.430 (2) & 1.434 (2) Å in (I)] deviate slightly from the normal mean value reported in the literature (Allen *et al.*, 1987). This indicates that the substitution of the phenylsulfonyl group at atom N1 results in an lengthening of the C—N bond lengths. This may be due to the electron-withdrawing character of the phenylsulfonyl group (Govindasamy *et al.*, 1998). The S atom exhibits significant deviation from that of a regular tetrahedron, with the largest deviations being seen for the O—S—O [O1—S1—O2 119.66 (7)°] and O—S—N angles [O1—S—N1 106.78 (7)°]. The widening of the angles may be due to repulsive interactions between the two short S=O bonds, similar to what is observed in related structures (Chakkaravarthi *et al.*, 2008; Rodriguez *et al.*, 1995).

The sum of the bond angles around N1 [350.99°] indicate the *sp*^2^ hybridized state of the atom N, in the molecule. The benzene ring C15—C20 is almost coplanar with methyl groups [torsion angles C24—C19—C20—C21 and C15—C16—C17—C23 (0.17 (30)° and -179.18 (22)°, respectively] The torsion angles O1—S1—N1—C7 and O1—S1—C1—C6 [43.11 (16)° and 22.47 (20)°, respectively] describe the *syn* conformation of the phenylsulfonyl group with respect to benzocarbazole ring system. This conformation is influenced by the intramolecular C—H··· O hydrogen bonds (Table 1), C8—H8··· O1 and C21—H21···O2, involving sulfonyl atoms O1 and O2.

In addition, intramolecular C8—H8···O1 and C21—H21···O2 hydrogen bonds form six-membered rings, both with a graph-set motif of S(6)  (Etter *et al.*, 1990). The crystal packing exhibits weak intermolecular C—H···π interactions, involving the C7—C12 (centroid *Cg*1) and C15—C20 (centroid *Cg*2) rings (Table 1) [Fig. 2].

### Experimental

To a solution of diethyl 2-((2-(bromomethyl)-1-(phenylsulfonyl)-1*H*-indol-3-yl)methylene) malonate (0.57 mmol) in dry 1,2-DCE (10 ml), ZnBr_2_ (1.15 mmol) and *m*-xylene (1.15 mmol) were added. The reaction mixture was then refluxed for 1 h under N_2_ atmosphere. It was then poured over ice-water (30 ml) containing 1 ml of concentrated HCl, extracted with chloroform (2 *X* 10 ml) and dried (Na_2_SO_4_). The solvent was removed under vacuo, then crude products was purified by flash column chromatography (silica gel, 230–420 mesh, n-hexane/ethyl acetate 99:1) afforded the compound (I), suitable for X-ray analysis.

### Refinement

H atoms were positioned geometrically and refined using riding model with C—H = 0.93 Å and *U*_iso_(H) = 1.2Ueq(C) for aromatic H atoms and C—H = 0.96 Å and *U*_iso_(H) = 1.5Ueq(C) for methyl H atoms.

### Crystal data

**Table:**

C_24_H_19_NO_2_S	*F*_000_ = 1616
*M**_r_* = 385.46	*D*_x_ = 1.320 Mg m^−^^3^
Orthorhombic, *P**b**c**a*	Mo *K*α radiation λ = 0.71073 Å
Hall symbol: -P 2ac 2ab	Cell parameters from 5907 reflections
*a* = 13.9260 (6) Å	θ = 2.6–28.4º
*b* = 10.1995 (5) Å	µ = 0.19 mm^−^^1^
*c* = 27.3014 (14) Å	*T* = 295 (2) K
*V* = 3877.8 (3) Å^3^	Block, colourless
*Z* = 8	0.30 × 0.20 × 0.16 mm

### Data collection

**Table:**

Bruker Kappa APEXII diffractometer	5626 independent reflections
Radiation source: fine-focus sealed tube	3490 reflections with *I* > 2σ(*I*)
Monochromator: graphite	*R*_int_ = 0.038
*T* = 295(2) K	θ_max_ = 30.0º
ω and φ scans	θ_min_ = 1.5º
Absorption correction: multi-scan(SADABS; Sheldrick, 1996)	*h* = −11→19
*T*_min_ = 0.940, *T*_max_ = 0.971	*k* = −14→11
26413 measured reflections	*l* = −37→38

### Refinement

**Table:**

Refinement on *F*^2^	Secondary atom site location: difference Fourier map
Least-squares matrix: full	Hydrogen site location: inferred from neighbouring sites
*R*[*F*^2^ > 2σ(*F*^2^)] = 0.051	H-atom parameters constrained
*wR*(*F*^2^) = 0.157	*w* = 1/[σ^2^(*F*_o_^2^) + (0.0716*P*)^2^ + 1.0437*P*] where *P* = (*F*_o_^2^ + 2*F*_c_^2^)/3
*S* = 1.04	(Δ/σ)_max_ < 0.001
5626 reflections	Δρ_max_ = 0.68 e Å^−^^3^
255 parameters	Δρ_min_ = −0.31 e Å^−^^3^
Primary atom site location: structure-invariant direct methods	Extinction correction: none

### Fractional atomic coordinates and isotropic or equivalent isotropic displacement parameters (Å^2^)

**Table:**

	*x*	*y*	*z*	*U*_iso_*/*U*_eq_	
S1	0.53043 (3)	0.01070 (5)	0.179632 (16)	0.04540 (15)	
O1	0.57705 (11)	0.10159 (16)	0.21087 (5)	0.0640 (4)	
O2	0.58196 (10)	−0.09761 (15)	0.16030 (5)	0.0577 (4)	
N1	0.49029 (11)	0.09650 (16)	0.13202 (5)	0.0426 (4)	
C1	0.42667 (14)	−0.0487 (2)	0.20902 (7)	0.0477 (5)	
C2	0.38759 (17)	−0.1665 (2)	0.19457 (8)	0.0591 (5)	
H2	0.4160	−0.2151	0.1696	0.071*	
C3	0.30584 (19)	−0.2116 (3)	0.21752 (10)	0.0772 (7)	
H3	0.2787	−0.2909	0.2079	0.093*	
C4	0.2648 (2)	−0.1411 (3)	0.25400 (11)	0.0809 (8)	
H4	0.2099	−0.1727	0.2694	0.097*	
C5	0.30312 (19)	−0.0243 (3)	0.26847 (10)	0.0785 (8)	
H5	0.2741	0.0228	0.2936	0.094*	
C6	0.38543 (18)	0.0250 (2)	0.24591 (8)	0.0627 (6)	
H6	0.4117	0.1049	0.2554	0.075*	
C7	0.44478 (13)	0.22122 (19)	0.13769 (7)	0.0454 (4)	
C8	0.46434 (16)	0.3208 (2)	0.17055 (8)	0.0581 (5)	
H8	0.5103	0.3106	0.1950	0.070*	
C9	0.4132 (2)	0.4357 (3)	0.16576 (9)	0.0717 (7)	
H9	0.4250	0.5042	0.1874	0.086*	
C10	0.3448 (2)	0.4517 (3)	0.12961 (10)	0.0745 (7)	
H10	0.3115	0.5304	0.1273	0.089*	
C11	0.32554 (17)	0.3529 (2)	0.09714 (9)	0.0643 (6)	
H11	0.2794	0.3642	0.0729	0.077*	
C12	0.37554 (14)	0.2355 (2)	0.10081 (7)	0.0486 (5)	
C13	0.37626 (13)	0.1181 (2)	0.07144 (6)	0.0446 (4)	
C14	0.32703 (14)	0.0819 (2)	0.02981 (7)	0.0497 (5)	
H14	0.2800	0.1367	0.0169	0.060*	
C15	0.34814 (13)	−0.0379 (2)	0.00694 (7)	0.0491 (5)	
C16	0.29952 (16)	−0.0775 (3)	−0.03650 (7)	0.0600 (6)	
H16	0.2513	−0.0243	−0.0492	0.072*	
C17	0.32182 (18)	−0.1911 (3)	−0.05996 (8)	0.0651 (7)	
C18	0.39480 (18)	−0.2716 (2)	−0.04030 (8)	0.0648 (6)	
H18	0.4103	−0.3488	−0.0566	0.078*	
C19	0.44376 (15)	−0.2408 (2)	0.00171 (7)	0.0546 (5)	
C20	0.42069 (14)	−0.12157 (19)	0.02648 (7)	0.0459 (4)	
C21	0.47023 (13)	−0.08330 (19)	0.06929 (6)	0.0435 (4)	
H21	0.5176	−0.1366	0.0827	0.052*	
C22	0.44720 (12)	0.03323 (19)	0.09060 (6)	0.0410 (4)	
C23	0.2711 (2)	−0.2323 (3)	−0.10647 (9)	0.0899 (9)	
H23A	0.2321	−0.1613	−0.1182	0.135*	
H23B	0.2312	−0.3070	−0.0999	0.135*	
H23C	0.3179	−0.2549	−0.1309	0.135*	
C24	0.5218 (2)	−0.3292 (2)	0.02088 (10)	0.0711 (7)	
H24A	0.5012	−0.3687	0.0510	0.107*	
H24B	0.5789	−0.2788	0.0266	0.107*	
H24C	0.5350	−0.3966	−0.0027	0.107*	

### Atomic displacement parameters (Å^2^)

**Table:**

	*U*^11^	*U*^22^	*U*^33^	*U*^12^	*U*^13^	*U*^23^
S1	0.0375 (2)	0.0617 (3)	0.0370 (2)	0.0086 (2)	−0.00775 (18)	0.0004 (2)
O1	0.0572 (9)	0.0790 (11)	0.0557 (9)	0.0017 (8)	−0.0242 (7)	−0.0081 (8)
O2	0.0462 (7)	0.0755 (10)	0.0514 (8)	0.0223 (7)	−0.0049 (6)	−0.0018 (7)
N1	0.0413 (8)	0.0515 (9)	0.0350 (7)	−0.0009 (7)	−0.0056 (6)	0.0016 (7)
C1	0.0475 (10)	0.0575 (12)	0.0381 (9)	0.0151 (9)	0.0005 (8)	0.0109 (8)
C2	0.0635 (13)	0.0623 (14)	0.0514 (11)	0.0022 (11)	0.0064 (10)	0.0072 (10)
C3	0.0754 (17)	0.0786 (17)	0.0776 (17)	−0.0049 (14)	0.0120 (14)	0.0157 (14)
C4	0.0658 (16)	0.095 (2)	0.0812 (18)	0.0141 (15)	0.0191 (14)	0.0270 (16)
C5	0.0709 (16)	0.100 (2)	0.0647 (15)	0.0353 (16)	0.0233 (13)	0.0080 (14)
C6	0.0665 (14)	0.0663 (14)	0.0553 (12)	0.0216 (11)	0.0047 (11)	0.0011 (11)
C7	0.0436 (9)	0.0517 (11)	0.0410 (9)	−0.0013 (8)	−0.0029 (8)	0.0032 (8)
C8	0.0616 (13)	0.0608 (13)	0.0518 (11)	0.0015 (11)	−0.0108 (10)	−0.0044 (10)
C9	0.0864 (17)	0.0596 (14)	0.0690 (15)	0.0060 (13)	−0.0117 (13)	−0.0124 (12)
C10	0.0851 (18)	0.0611 (15)	0.0773 (17)	0.0192 (13)	−0.0111 (14)	−0.0043 (13)
C11	0.0618 (13)	0.0677 (15)	0.0635 (14)	0.0129 (11)	−0.0145 (11)	0.0051 (12)
C12	0.0450 (10)	0.0561 (12)	0.0446 (10)	0.0007 (9)	−0.0052 (8)	0.0056 (9)
C13	0.0387 (9)	0.0575 (11)	0.0376 (9)	−0.0068 (8)	−0.0033 (7)	0.0085 (8)
C14	0.0437 (10)	0.0622 (12)	0.0433 (10)	−0.0063 (9)	−0.0102 (8)	0.0104 (9)
C15	0.0440 (10)	0.0675 (13)	0.0359 (9)	−0.0200 (9)	−0.0030 (8)	0.0083 (9)
C16	0.0556 (12)	0.0824 (16)	0.0422 (10)	−0.0268 (11)	−0.0098 (9)	0.0062 (11)
C17	0.0692 (14)	0.0837 (17)	0.0425 (11)	−0.0398 (13)	−0.0033 (10)	−0.0006 (11)
C18	0.0757 (15)	0.0689 (15)	0.0499 (11)	−0.0344 (12)	0.0056 (11)	−0.0080 (11)
C19	0.0628 (12)	0.0549 (12)	0.0462 (10)	−0.0237 (10)	0.0041 (9)	0.0006 (9)
C20	0.0479 (10)	0.0532 (11)	0.0366 (9)	−0.0171 (8)	0.0035 (8)	0.0041 (8)
C21	0.0446 (10)	0.0500 (10)	0.0358 (9)	−0.0065 (8)	−0.0017 (7)	0.0058 (8)
C22	0.0381 (9)	0.0522 (11)	0.0329 (8)	−0.0072 (8)	−0.0026 (7)	0.0051 (7)
C23	0.099 (2)	0.117 (2)	0.0532 (13)	−0.0450 (19)	−0.0173 (13)	−0.0105 (15)
C24	0.0975 (19)	0.0495 (13)	0.0662 (15)	−0.0065 (13)	0.0034 (13)	−0.0067 (11)

### Geometric parameters (Å, °)

**Table:**

S1—O1	1.4170 (15)	C11—H11	0.9300
S1—O2	1.4190 (15)	C12—C13	1.442 (3)
S1—N1	1.6636 (15)	C13—C14	1.377 (3)
S1—C1	1.760 (2)	C13—C22	1.414 (3)
N1—C7	1.430 (2)	C14—C15	1.404 (3)
N1—C22	1.434 (2)	C14—H14	0.9300
C1—C2	1.377 (3)	C15—C16	1.424 (3)
C1—C6	1.382 (3)	C15—C20	1.426 (3)
C2—C3	1.379 (3)	C16—C17	1.360 (4)
C2—H2	0.9300	C16—H16	0.9300
C3—C4	1.355 (4)	C17—C18	1.412 (4)
C3—H3	0.9300	C17—C23	1.513 (3)
C4—C5	1.363 (4)	C18—C19	1.371 (3)
C4—H4	0.9300	C18—H18	0.9300
C5—C6	1.395 (4)	C19—C20	1.428 (3)
C5—H5	0.9300	C19—C24	1.506 (3)
C6—H6	0.9300	C20—C21	1.412 (3)
C7—C8	1.382 (3)	C21—C22	1.362 (3)
C7—C12	1.402 (3)	C21—H21	0.9300
C8—C9	1.378 (3)	C23—H23A	0.9600
C8—H8	0.9300	C23—H23B	0.9600
C9—C10	1.381 (4)	C23—H23C	0.9600
C9—H9	0.9300	C24—H24A	0.9600
C10—C11	1.368 (3)	C24—H24B	0.9600
C10—H10	0.9300	C24—H24C	0.9600
C11—C12	1.388 (3)		
			
O1—S1—O2	120.10 (9)	C7—C12—C13	107.97 (17)
O1—S1—N1	106.26 (9)	C14—C13—C22	119.30 (19)
O2—S1—N1	106.79 (8)	C14—C13—C12	132.68 (19)
O1—S1—C1	109.07 (10)	C22—C13—C12	107.91 (16)
O2—S1—C1	108.47 (10)	C13—C14—C15	119.73 (18)
N1—S1—C1	105.14 (8)	C13—C14—H14	120.1
C7—N1—C22	107.48 (14)	C15—C14—H14	120.1
C7—N1—S1	122.17 (12)	C14—C15—C16	121.2 (2)
C22—N1—S1	121.34 (13)	C14—C15—C20	120.20 (17)
C2—C1—C6	121.3 (2)	C16—C15—C20	118.6 (2)
C2—C1—S1	119.61 (16)	C17—C16—C15	121.7 (2)
C6—C1—S1	119.11 (19)	C17—C16—H16	119.2
C1—C2—C3	119.2 (2)	C15—C16—H16	119.2
C1—C2—H2	120.4	C16—C17—C18	118.7 (2)
C3—C2—H2	120.4	C16—C17—C23	121.7 (3)
C4—C3—C2	120.3 (3)	C18—C17—C23	119.5 (3)
C4—C3—H3	119.8	C19—C18—C17	122.9 (2)
C2—C3—H3	119.8	C19—C18—H18	118.6
C3—C4—C5	120.8 (3)	C17—C18—H18	118.6
C3—C4—H4	119.6	C18—C19—C20	118.6 (2)
C5—C4—H4	119.6	C18—C19—C24	120.8 (2)
C4—C5—C6	120.6 (2)	C20—C19—C24	120.51 (19)
C4—C5—H5	119.7	C21—C20—C15	119.36 (18)
C6—C5—H5	119.7	C21—C20—C19	121.15 (19)
C1—C6—C5	117.9 (3)	C15—C20—C19	119.46 (18)
C1—C6—H6	121.1	C22—C21—C20	118.67 (18)
C5—C6—H6	121.1	C22—C21—H21	120.7
C8—C7—C12	121.70 (19)	C20—C21—H21	120.7
C8—C7—N1	129.54 (17)	C21—C22—C13	122.74 (16)
C12—C7—N1	108.65 (17)	C21—C22—N1	129.15 (17)
C9—C8—C7	117.5 (2)	C13—C22—N1	107.99 (16)
C9—C8—H8	121.3	C17—C23—H23A	109.5
C7—C8—H8	121.3	C17—C23—H23B	109.5
C8—C9—C10	121.6 (2)	H23A—C23—H23B	109.5
C8—C9—H9	119.2	C17—C23—H23C	109.5
C10—C9—H9	119.2	H23A—C23—H23C	109.5
C11—C10—C9	120.7 (2)	H23B—C23—H23C	109.5
C11—C10—H10	119.6	C19—C24—H24A	109.5
C9—C10—H10	119.6	C19—C24—H24B	109.5
C10—C11—C12	119.3 (2)	H24A—C24—H24B	109.5
C10—C11—H11	120.3	C19—C24—H24C	109.5
C12—C11—H11	120.3	H24A—C24—H24C	109.5
C11—C12—C7	119.10 (19)	H24B—C24—H24C	109.5
C11—C12—C13	132.82 (18)		
			
O1—S1—N1—C7	−43.11 (16)	C7—C12—C13—C14	−176.6 (2)
O2—S1—N1—C7	−172.43 (14)	C11—C12—C13—C22	175.6 (2)
C1—S1—N1—C7	72.46 (16)	C7—C12—C13—C22	−0.6 (2)
O1—S1—N1—C22	173.90 (14)	C22—C13—C14—C15	−0.3 (3)
O2—S1—N1—C22	44.58 (16)	C12—C13—C14—C15	175.41 (19)
C1—S1—N1—C22	−70.53 (16)	C13—C14—C15—C16	−179.16 (17)
O1—S1—C1—C2	−158.20 (16)	C13—C14—C15—C20	−0.4 (3)
O2—S1—C1—C2	−25.75 (18)	C14—C15—C16—C17	177.57 (19)
N1—S1—C1—C2	88.19 (17)	C20—C15—C16—C17	−1.2 (3)
O1—S1—C1—C6	22.46 (19)	C15—C16—C17—C18	0.4 (3)
O2—S1—C1—C6	154.91 (16)	C15—C16—C17—C23	−179.2 (2)
N1—S1—C1—C6	−91.15 (17)	C16—C17—C18—C19	0.6 (3)
C6—C1—C2—C3	−0.1 (3)	C23—C17—C18—C19	−179.9 (2)
S1—C1—C2—C3	−179.44 (18)	C17—C18—C19—C20	−0.6 (3)
C1—C2—C3—C4	−0.4 (4)	C17—C18—C19—C24	−179.3 (2)
C2—C3—C4—C5	0.4 (4)	C14—C15—C20—C21	0.7 (3)
C3—C4—C5—C6	0.0 (4)	C16—C15—C20—C21	179.44 (17)
C2—C1—C6—C5	0.5 (3)	C14—C15—C20—C19	−177.66 (17)
S1—C1—C6—C5	179.85 (17)	C16—C15—C20—C19	1.1 (3)
C4—C5—C6—C1	−0.5 (4)	C18—C19—C20—C21	−178.54 (18)
C22—N1—C7—C8	−176.3 (2)	C24—C19—C20—C21	0.2 (3)
S1—N1—C7—C8	36.3 (3)	C18—C19—C20—C15	−0.2 (3)
C22—N1—C7—C12	−0.2 (2)	C24—C19—C20—C15	178.47 (18)
S1—N1—C7—C12	−147.55 (14)	C15—C20—C21—C22	−0.2 (3)
C12—C7—C8—C9	−0.1 (3)	C19—C20—C21—C22	178.10 (16)
N1—C7—C8—C9	175.6 (2)	C20—C21—C22—C13	−0.5 (3)
C7—C8—C9—C10	0.0 (4)	C20—C21—C22—N1	−176.05 (17)
C8—C9—C10—C11	0.0 (4)	C14—C13—C22—C21	0.8 (3)
C9—C10—C11—C12	0.0 (4)	C12—C13—C22—C21	−175.89 (17)
C10—C11—C12—C7	−0.1 (3)	C14—C13—C22—N1	177.13 (16)
C10—C11—C12—C13	−175.9 (2)	C12—C13—C22—N1	0.5 (2)
C8—C7—C12—C11	0.2 (3)	C7—N1—C22—C21	175.86 (18)
N1—C7—C12—C11	−176.30 (18)	S1—N1—C22—C21	−36.4 (2)
C8—C7—C12—C13	176.93 (19)	C7—N1—C22—C13	−0.19 (19)
N1—C7—C12—C13	0.5 (2)	S1—N1—C22—C13	147.52 (13)
C11—C12—C13—C14	−0.5 (4)		

### Hydrogen-bond geometry (Å, °)

**Table:**

*D*—H···*A*	*D*—H	H···*A*	*D*···*A*	*D*—H···*A*
C8—H8···O1	0.93	2.37	2.945 (3)	120
C21—H21···O2	0.93	2.34	2.935 (2)	122
C8—H8···Cg1^i^	0.93	3.00	3.859	155
C11—H11···Cg2^ii^	0.93	2.90	3.693	144

Symmetry codes: (i) −*x*+1, *y*+1/2, −*z*+1/2; (ii) −*x*−1/2, *y*−1/2, *z*.
